# Phylogeography of *Francisella tularensis *subspecies *holarctica *from the country of Georgia

**DOI:** 10.1186/1471-2180-11-139

**Published:** 2011-06-17

**Authors:** Gvantsa Chanturia, Dawn N Birdsell, Merab Kekelidze, Ekaterine Zhgenti, George Babuadze, Nikoloz Tsertsvadze, Shota Tsanava, Paata Imnadze, Stephen M Beckstrom-Sternberg, James S Beckstrom-Sternberg, Mia D Champion, Shripad Sinari, Miklos Gyuranecz, Jason Farlow, Amanda H Pettus, Emily L Kaufman, Joseph D Busch, Talima Pearson, Jeffrey T Foster, Amy J Vogler, David M Wagner, Paul Keim

**Affiliations:** 1 Center for Microbial Genetics and Genomics, Northern Arizona University, Flagstaff, AZ 86011-4073, USA; 2 National Center for Disease Control and Public Health, Tbilisi, 0177, Georgia; 3 Translational Genomics Research Institute, Phoenix, AZ 85004, USA; 4 Veterinary Medical Research Institute, Hungarian Academy of Sciences, Budapest, Hungary; 5 US Army Medical Research Institute of Infectious Diseases, Fort Detrick, Frederick, Maryland 21702-5011, USA

## Abstract

**Background:**

*Francisella tularensis*, the causative agent of tularemia, displays subspecies-specific differences in virulence, geographic distribution, and genetic diversity. *F. tularensis *subsp. *holarctica *is widely distributed throughout the Northern Hemisphere. In Europe, *F. tularensis *subsp. *holarctica *isolates have largely been assigned to two phylogenetic groups that have specific geographic distributions. Most isolates from Western Europe are assigned to the B.Br.FTNF002-00 group, whereas most isolates from Eastern Europe are assigned to numerous lineages within the B.Br.013 group. The eastern geographic extent of the B.Br.013 group is currently unknown due to a lack of phylogenetic knowledge about populations at the European/Asian juncture and in Asia. In this study, we address this knowledge gap by describing the phylogenetic structure of *F. tularensis *subsp. *holarctica *isolates from the country of Georgia, and by placing these isolates into a global phylogeographic context.

**Results:**

We identified a new genetic lineage of *F. tularensis *subsp. *holarctica *from Georgia that belongs to the B.Br.013 group. This new lineage is genetically and geographically distinct from lineages previously described from the B.Br.013 group from Central-Eastern Europe. Importantly, this new lineage is basal within the B.Br.013 group, indicating the Georgian lineage diverged before the diversification of the other known B.Br.013 lineages. Although two isolates from the Georgian lineage were collected nearby in the Ukrainian region of Crimea, all other global isolates assigned to this lineage were collected in Georgia. This restricted geographic distribution, as well as the high levels of genetic diversity within the lineage, is consistent with a relatively older origin and localized differentiation.

**Conclusions:**

We identified a new lineage of *F. tularensis *subsp. *holarctica *from Georgia that appears to have an older origin than any other diversified lineages previously described from the B.Br.013 group. This finding suggests that additional phylogenetic studies of *F. tularensis *subsp. *holarctica *populations in Eastern Europe and Asia have the potential to yield important new insights into the evolutionary history and phylogeography of this broadly dispersed *F. tularensis *subspecies.

## Background

*Francisella tularensis *is a highly clonal, recently-emerged pathogen that causes tularemia, which presents in several main forms: pneumonic (30%-60% mortality), ulceroglandular, and oropharyngeal [1]. The latter two are associated with lower mortality. *F. tularensis *is currently divided into three subspecies (*tularensis*, *holarctica *and *mediasiatica*), with *F. novicida *recognized as a very closely related species, or as another subspecies by some authors [2-4]. These taxa vary in virulence, geographic distribution, overall genetic diversity, and host/vector associations [3,5-9]. Human tularemia is a disease at which the clinical severity depends upon the route of infection, subspecies of the infection strain, and timely therapeutic response [9]. Cases in Europe are caused by *F. tularensis *subsp. *holarctica*, and in many rural areas of the Balkans and countries further east outbreaks are water-borne, resulting in oropharyngeal tularemia [10-12]. No known cases by *F. tularensis *subsp. *mediasiatica *are known and only a few by *F. novicida *have been documented [13,14]. *F. tularensis *subsp. *tularensis *is restricted to North America, whereas *F. tularensis *subsp. *holarctica *is found throughout the Northern Hemisphere [3,15]. Despite its wider geographic distribution *F. tularensis *subsp. *holarctica *has markedly lower genetic diversity than *F. tularensis *subsp. *tularensis *[5,7,8].

**Figure 1:**
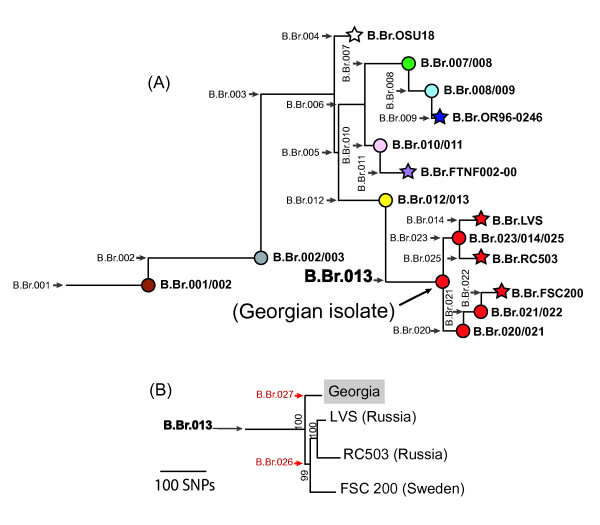
**Phylogenies of *Francisella tularensis *subsp. *holarctica***. (A) CanSNP phylogeny of *Francisella tularensis *subsp. *holarctica *subclades identified by Vogler et al. and Svensson et al. [15,16] (See additional file 1 for an update of these SNP positions based on the latest SCHU S4 genome NC_006570). Subclades within the B.Br.013 group are depicted in red. The Georgian isolate was placed in the basal node B.Br.013/020/023 (black arrow). (B) Maximum parsimony SNP phylogeny of four *F. tularensis *whole genome sequences from the B.Br.013 group. The Georgian strain is highlighted in gray and is basal to the other three genomes. Newly identified branches (B.Br.027 and B.Br.026) are colored red and showed two major divisions within the B.Br.013 group. This phylogeny was rooted using OSU18 (not depicted). Bootstrap values are based on 1000 replicates in PAUP using a heuristic search.

Significant gains toward deciphering the evolutionary history of *F. tularensis *overall and, in particular, *F. tularensis *subsp. *holarctica *have been made by using whole genome comparisons for single nucleotide polymorphism (SNP) discovery coupled with subsequent canonical SNP (canSNP) analysis [15,16]. Numerous new groups were identified within *F. tularensis *subsp. *holarctica *(Figure 1A) [15,16], two of which, B.Br.013 (includes subclades B.Br.013/014 and B.Br.LVS in [15]) and B.Br.FTNF002-00, were predominant in Europe but geographically segregated [15]. In the Western European countries of Spain, France, and Switzerland almost all isolates belong to the highly monomorphic B.Br.FTNF002-00 group [15-18]. In contrast, in large portions of Central and Eastern Europe, from the Czech Republic to Russia, most *F. tularensis *subsp. *holarctica *isolates are assigned to various lineages within the B.Br.013 group [15,16].

Additional analyses of the B.Br.013 group are crucial for fully understanding the phylogeography of *F. tularensis *subsp. *holarctica *in Europe and Asia. This group contains significant genetic diversity based upon multi-locus variable-number tandem repeat (VNTR) analysis (MLVA) [15], indicating that considerable phylogenetic structure may exist that could be revealed with additional analyses. In addition, this group is widely distributed, extending from Eastern Europe into the border regions of the European/Asian continents. Importantly, the eastern geographic extent of the B.Br.013 group is very poorly understood. This is because, to date, it has not been possible to place *F. tularensis *isolates from countries at the boundary of the European/Asian continents and Western Asia, including Georgia, into a larger phylogeographic context. Based on growth characteristics, biochemical analyses, basic PCR methods, and DNA sequencing, we know that *F. tularensis *subsp. *holarctica *is the predominant subspecies in Georgia and in regions further east [11,19-21], but more specific genetic information is limited. Some isolates from the European/Asian juncture regions and East Asia have been genotyped with a subset of VNTRs but have not been part of any global analyses [10,22,23]. Although valuable for regional studies, homoplasy associated with these rapidly-evolving markers restricts their value for global phylogenetic analyses [24].

In this study, we determined the phylogenetic structure of *F. tularensis *subsp. *holarctica *isolates from the European/Asian juncture country of Georgia by sequencing the genome of a Georgian isolate, comparing that genome to other available whole genome sequences to discover SNPs, and screening a subset of the resulting SNPs across 25 isolates from Georgia. We examined diversity within the subclades defined by these SNPs using a multiple-locus variable number tandem repeat analysis (MLVA) system [25]. To place the Georgian isolates into an existing global phylogeographic framework [15], we also screened a canonical subset of the newly discovered SNPs across a large panel of European isolates belonging to the B.Br.013 group.

## Results

### Georgian isolate whole genome sequence

Initial analyses with previously described canSNP assays (See Additional file 1, [15]) revealed that all 25 Georgian isolates belong to the B.Br.013 group. One of the Georgian strains (F0673) was sequenced using the Illumina Genome Analyzer II sequencing platform resulting in very high sequence coverage (averaging 1,076X) when aligned to the LVS genome (See Additional file 2, [26]). Subsequent whole genome sequence (WGS) comparisons among three published B.Br.013 group genomes (FSC 200, LVS, and RC503), the genome of strain F0673 generated for this study, and the published OSU18 genome (as an outgroup) revealed 650 putative SNPs. Most of these putative SNPs (n = 470) were phylogenetically located on the branches separating OSU18 from the genomes in the B.Br.013 group (data not shown). Maximum parsimony analysis of the putative SNPs produced a phylogeny (Figure 1B) with a very low homoplasy index (0.02), consistent with the highly clonal nature of *F. tularensis*. The phylogenetic topology of the FSC 200, LVS, and RC503 genomes is consistent with previous publications [15,16], and the small number of putative SNPs unique to the Georgian strain is consistent with the low genetic diversity observed among other lineages within *F. tularensis *subsp. *holarctica *[3,6,27,28]. The new branch (B.Br.027) leading to the Georgian strain arises from a common ancestor that is basal to the previously described diversified lineages within the B.Br.013 group and is separated from them by only 45 putative SNPs, with 39 of these putative SNPs leading to the Georgian strain (B.Br.027 in Figure 1B) and the other six putative SNPs along a branch (B.Br.026 in Figure 1B) defining a monophyletic lineage containing the other sequenced strains from this group.

### Identification of new lineages and subclades

**Figure 2:**
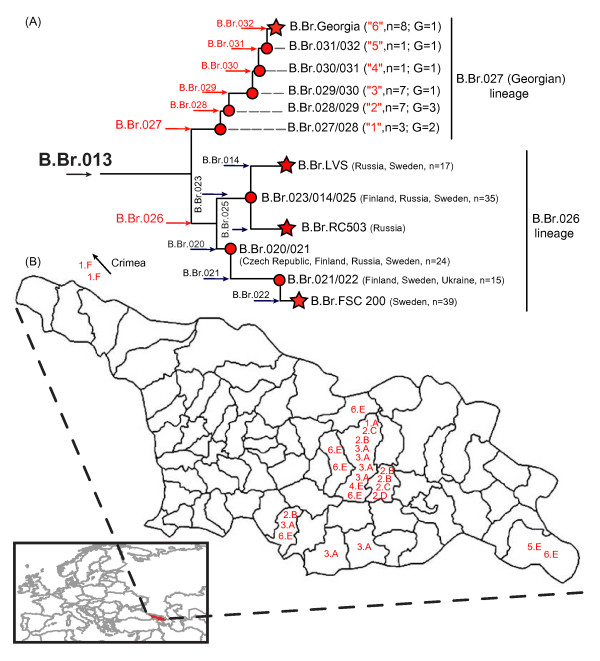
**Subclade phylogeny and geographic distribution**. (A) CanSNP phylogeny of the Georgian subclades within the Br.013 group. Terminal subclades representing sequenced strains are shown as stars and intervening nodes representing collapsed branches are indicated by circles. Newly identified branches are indicated in red and previously published branches are indicated in black. The right vertical black bars indicate the subclades that comprise the two major lineages within the B.Br.013 group. The number of isolates (n), MLVA genotypes (G), and a number in quotations to digitally represent each Georgian subclade on the distribution map. Dashes (- -) indicate hypothetical branch lengths for collapsed nodes. (B) Distribution of Georgian lineage subclades in the country of Georgia. The global geographic map indicates Georgia colored as red (lower left) and dashed lines show an enlarged map of Georgia at the district scale. Subclade and MLVA genotypes for each isolate are shown alphanumerically. The number corresponds to subclade designations in the expanded Georgian (B.Br.027) lineage of the B.Br.013 group phylogenetic tree in (A), and the letter corresponds to MLVA genotypes indicated in Table 2 and in Additional file 4. Subclade and MLVA genotypes are also shown for the two Crimean isolates, indicated by an arrow pointing in the direction of the Crimean peninsula (upper left).

We designed assays targeting 21 of the 39 putative SNPs leading to the sequenced Georgian strain (Table 1) and screened them across the 25 Georgian isolates (Table 2) to reveal additional phylogenetic structure among these strains. All 21 SNPs were determined to be real and assigned the 25 strains to a monophyletic lineage (B.Br.027; also referred to below as the Georgian lineage) that includes six new subclades (Figure 2A). We also designed an assay (Table 1) targeting one of six putative SNPs along the branch (B.Br.026 in Figure 1B) leading to the other sequenced strains (FSC 200, LVS, and RC503) and screened it across DNA extracts from these three sequenced strains, as well as the 25 strains in the Georgian lineage. Consistent with the bioinformatics analyses, DNA extracts from the three sequenced strains all possessed the derived state for this SNP, whereas the 25 strains in the Georgian lineage all possessed the ancestral state for this SNP. This confirmed that the SNP was real and also branch B.Br.026, which leads to the lineage that gave rise to the previously known subclades within the B.Br.013 group [16]. Altogether, we identified a total of 7 new branches (B.Br.026-B.Br.032, Figure 2A) and designated a single canSNP for each of these branches with corresponding SNP genotyping assays (Table 1). Designating a single SNP as canonical for each branch maximizes phylogenetic information while minimizing the number of required assays by eliminating redundant SNPs, thus providing a highly efficient means of determining the phylogenetic positions of isolates for highly clonal pathogens such as *F. tularensis *[15,24]. In addition, canSNPs represent standardized phylogenetic positions for comparison in future studies performed by different research groups.

**Table 1:** Melt-MAMA primers targeting informative canSNPs

SNP	SCHU S4 position	Genome SNP state (D/A)^*a*^	Melt MAMA primer^*c*^	Melt-MAMA primer sequences^*d*^	Primer conc. (μM)	Annealing temp. (°C)	Melting T_m _(°C)
B.Br.026	1484645	A/C	D	GAAACTTATTTGTTCCTAAGACAGTGACAcTA	0.800	55	73.1
			A	ggggcggggcggggcAAACTTATTTGTTCCTAAGACAGTGACAgTC	0.200		79.7
			C	GCATTGAGTTTGACAGGGTTGC	0.200		

B.Br.027	1329722	T/G^*b*^	D	ggggcggggcggggcggggcCATGCCAGGCACTACAATTGATAGTaTA	0.200	55	78.2
			A	TGCCAGGCACTACAATTGATAGTtTC	1.000		73.6
			C	TATACTTCTGACCATGGCGTTCAAAT	0.200		

B.Br.028	212729	T/G	D	ggggcggggcggggcggggcAAATTAGTTCAAATGTTAAATTTGATcCT	0.200	55	75.8
			A	AAATTAGTTCAAATGTTAAATTTGATaCG	0.200		67.7
			C	CAAAATAAATCCCGTTGAGAATAGAA	0.200		

B.Br.029	1185519	A/G	D	ggggcggggcggggcggggcTGCTTAATCTCATTGACTAGCTGTGgTA	0.200	55	78
			A	TGCTTAATCTCATTGACTAGCTGTGaTG	1.000		70
			C	ACAAAGTTGAAACTATCGAGCATAAATC	0.200		

B.Br.030	928335	T/G	D	ggggcggggcggggcggggcTGTTGGGTCAAAGAGAGAAGTgTT	0.200	55	78.2
			A	ATTGTTGGGTCAAAGAGAGAAGTaTG	0.200		70
			C	GCCACCAAAGAATACAGAGTAGTCAT	0.200		

B.Br.031	1634565	A/G	D	ggggcggggcggggcggggcGCACCAATCGTATCTAATTGATcCA	0.400	55	79
			A	GCACCAATCGTATCTAATTGATtCG	0.200		70
			C	AACTTTGCTAAAACAAATGCTGTTGC	0.200		

B.Br.032	283540	A/G^*b*^	D	ggggcggggcggggcggggcTGCTAAACCTACAGTAATCAGAAGTATtAT	0.200	55	72
			A	TGCTAAACCTACAGTAATCAGAAGTATcAC	0.600		68.4
			C	GCTAAATTTTAGTAAGATAAAAAGTGTAAGTAGTG	0.200		

^*a*^SNP states are presented according to their orientation in the SCHU S4 reference genome (NC_006570);

^*b*^Assays designed from the reverse complement of the reference sequence.

^*c*^D: Derived; A: Ancestral; C: Common Primer

^*d*^Primer tails and antepenultimate mismatch bases are in lower case

**Table 2:** *Francisella tularensis *subsp. *holarctica *isolates from the country of Georgia used in this study.

ID^*a*^	State/Province	County/Region	Location^*b*^	Source	Date	SNP Subclade^*c*^	MLVA Genotype^*d*^
F0677	Shida Kartli	Gori	village Lamiskana	*Haemaphysalis otophila*	03/00/2008	B.Br.027/028	A
F0658	Shida Kartli	Kaspi	village Rene	water	00/00/2007	B.Br.028/029	B
F0660	Shida Kartli	Gori	village Nadarbazevi	*Dermacentor marginatus*	00/00/2004	B.Br.028/029	C
F0662	Samtskhe-Javakheti	Akhaltsikhe	village Minadze	fleas	00/00/1997	B.Br.028/029	B
F0674	Shida Kartli	Kaspi	village Rene	*Dermacentor marginatus*	04/00/2007	B.Br.028/029	B
F0675	Shida Kartli	Gori	village Nadarbazevi	*Haemaphysalis otophila*	04/00/2007	B.Br.028/029	B
F0678	Shida Kartli	Kaspi	village z/Rene	*Dermacentor marginatus*	06/00/2008	B.Br.028/029	C
F0679	Shida Kartli	Kaspi	village z/Rene	*Haemaphysalis sulcata*	06/00/2008	B.Br.028/029	D
F0659	Kvemo Kartli	Dmanisi	unknown	*Microtus arvalis Pall*.	00/00/1990	B.Br.029/030	A
F0665	Shida Kartli	Gori	village Shavshvebi	*Gamasidae *ticks	00/00/1982	B.Br.029/030	A
F0666	Samtskhe-Javakheti	Aspindza	village Indusa	*Dermacentor marginatus*	00/00/2004	B.Br.029/030	A
F0667	Shida Kartli	Gori	village Nadarbazevi	*Dermacentor marginatus*	00/00/2004	B.Br.029/030	A
F0668	Shida Kartli	Gori	village Nadarbazevi	*Dermacentor marginatus*	00/00/2004	B.Br.029/030	A
F0669	Samtskhe-Javakheti	Ninotsminda	unknown	*Dermacentor marginatus*	00/00/2002	B.Br.029/030	A
F0670	Shida Kartli	Gori	village Tkviavi	*Dermacentor marginatus*	00/00/2004	B.Br.029/030	A
F0672	Shida Kartli	Gori	village Khurvaleti	*Dermacentor marginatus*	00/00/2004	B.Br.030/031	E
F0655	Kakheti	Dedoplis Tskaro	Solukh steppe	*Meriones erythrurus *Gray	00/00/1956	B.Br.031/032	E
F0656	Kakheti	Dedoplis Tskaro	Nazarlebi Mountain	*Ixodidae *tick	00/00/1956	B.Br.Georgia	E
F0657	Shida Kartli	Tskhinvali	village Khetagurov	*Sorex *sp.	00/00/1974	B.Br.Georgia	E
F0661	Samtskhe-Javakheti	Akhaltsikhe	village Klde	*Microtus socialis Pall*.	00/00/1992	B.Br.Georgia	E
F0663	Shida Kartli	Kareli	village Ruisi	*Ixodidae *tick	00/00/1997	B.Br.Georgia	E
F0664	Shida Kartli	Kareli	village Ruisi	wheat	00/00/1997	B.Br.Georgia	E
F0671	unknown	unknown	East Georgia	unknown	unknown	B.Br.Georgia	E
F0673	unknown	unknown	East Georgia	unknown	unknown	B.Br.Georgia	E
F0676	Shida Kartli	Gori	village Nadarbazevi	*Dermacentor marginatus*	05/00/2007	B.Br.Georgia	E

^*a*^Strain ID in the Northern Arizona University DNA collection

^*b*^City, Town, or Village

^*c*^canSNP lineage

^*d*^Genotypes (A to E) determined by MLVA11 system (Vogler et al, 2009).

To understand the relationship of the Georgian lineage to other Eastern European lineages, we genotyped 132 geographically diverse group B.Br.013 isolates collected in Central and Eastern Europe across the B.Br.026 and B.Br.027 canSNP assays (Figure 2A, see additional file 3). All resulting genotypes from this analysis were phylogenetically consistent with no observed homoplasy. With just two exceptions, all of these isolates were assigned to the B.Br.026 lineage. The exceptions were two isolates from the Crimean region of Ukraine that were assigned to the Georgian lineage. Subsequent, additional canSNP analyses assigned these two isolates to the basal B.Br.027/028 subclade within the Georgian lineage. These results indicate that the Georgian isolates, as well as the two isolates from Crimea, are phylogenetically distinct from the previously described *F. tularensis *subsp. *holarctica *subpopulations.

The subclades within the Georgian lineage did not display a differentiated phylogeographic pattern but, rather, were spatially dispersed in a mixed fashion throughout Eastern Georgia and the Crimean region of Ukraine (Figure 2B). The assignment of the Crimean isolates to the basal B.Br.027/028 subclade within the Georgian lineage (Figure 2A) confirms that this lineage is not geographically restricted to Georgia, and is suggestive of a north to south dispersal pattern. That said, the overall geographic extent of the Georgian lineage is currently unknown due to the limited sampling in adjacent countries.

### Further discrimination using MLVA

MLVA was used to examine genetic variation within each identified subclade of the Georgian lineage (Table 2; Additional file 4). Five unique MLVA genotypes were identified among the 25 Georgian isolates (Table 2) that were distinct from the MLVA genotypes of strains found north of Georgia. Calculations of MLVA diversity (D = G/N) within each subclade (see methods for calculation) showed decreasing levels of diversity within higher resolution subclades (Figure 2A). The most basal Georgian subclade, B.Br.027/028 (D = 0.67) (Figure 2A), was comprised of a single Georgian isolate that was distinguishable from the two Crimean isolates in the same subclade due to a distinct MLVA genotype. There were three MLVA genotypes among the seven Georgian isolates within subclade B.Br.028/029 (D = 0.43). A single MLVA genotype was shared by all seven Georgian isolates in subclade B.Br.029/030 (D = 0.14), and the two other intermediate subclades (B.Br.030/031 and B.Br.031/032) contained only a single isolate each. Only a single MLVA genotype was observed among these two isolates and the eight isolates in the terminal subclade B.Br.Georgia (D = 0.13 in subclade B.Br.Georgia) (Figure 2A, Table 2). In general, MLVA diversity trended towards lower values nearer to the branch tip, consistent with shorter evolutionary times to generate diversity.

## Discussion

The low number of SNPs found globally among *F. tularensis *subsp. *holarctica *isolates suggests that this subspecies only recently emerged through a genetic bottleneck and then rapidly dispersed across the Northern Hemisphere [3,7,8,29,30]. The phylogeographic model of Vogler et al. [15] suggests a North American derivation for the main *F. tularensis *subsp. *holarctica *radiation that spread throughout the Northern Hemisphere. However, previous analyses of the spread throughout Europe and Asia were hindered by a lack of isolates from the regions along the European/Asian juncture and in East Asia. This study begins to address this knowledge gap by describing additional phylogenetic structure based upon 25 isolates from the European/Asian border country of Georgia through the use of SNPs discovered from whole genome comparisons.

Whole genome sequencing of a Georgian strain revealed SNPs that placed the Georgian lineage basal to the diversification of the subclades of the B.Br.026 lineage within the B.Br.013 group [15,16] (Figure 1B). In addition, a relatively large number of subclades (phylogenetic topology) within the Georgian lineage were discovered amongst a relatively small number of Georgian isolates. This is fortuitous, and perhaps a consequence of the selection of Georgian strain F0673 for sequencing [31,32].

Georgian (B.Br.027) lineage isolates are geographically distinct from the B.Br.026 lineage isolates. Georgian lineage isolates appear restricted to regions of the Ukraine and Georgia, whereas the B.Br.026 lineage isolates are concentrated in Central-Eastern Europe, based upon the isolates examined here. However, the true geographic extent of the Georgian lineage could not be fully determined due to the lack of a comprehensive set of isolates from regions neighboring Georgia. That said, it is clear that the Georgian lineage is absent from Central Europe. The geographic division of the B.Br.013 and B.Br.FTNF002-00 groups into Eastern and Western Europe, respectively, suggests that the common ancestor to these two lineages, and possibly the Georgian and north of Georgia lineages (B.Br.027 and B.Br.026, respectively), existed west of Georgia, although the lack of a comprehensive set of Asian isolates limits our ability to draw conclusions about the *F. tularensis *subsp. *holarctica *radiation that spread throughout Eurasia. Likewise, data from our current collection of isolates suggest that *F. tularensis *was introduced into Georgia from the north, though we unfortunately lack comparable isolates from the Middle East. For the entire *F. tularensis *subsp. *holarctica *radiation in Eurasia, a Scandinavian origin remains the most robust hypothesis given that Sweden contains the most phylogenetically diverse set of isolates in Eurasia, including isolates found in the subclade immediately basal to the B.Br.013 group [15].

Interestingly, at this regional scale, canSNPs and MLVA exhibited considerable congruence in identifying genetic groups. Specifically, canSNPs identified six subclades and MLVA identified five, albeit with slightly different but not phylogenetically inconsistent membership due to the nature of the two different marker types. SNPs discovered from whole genome sequences will typically provide greater discrimination than MLVA, as seen in subclades B.Br.030/031, B.Br.031/032 and B.Br.Georgia (Table 2), and can even be used to identify specific strains [33]. However, discovering these rare SNPs requires whole genome sequencing whereas MLVA can identify nearly the same number of genetic groups by simply surveying a few highly polymorphic portions of the genome. At this regional scale, homoplasy does not appear to be much of a factor in obscuring phylogenetic signal for identifying genetic groups using MLVA, although the relationships among those groups are less resolved as isolates from adjacent groups share MLVA genotypes. Together, SNPs and MLVA provide complementary approaches, by first accurately placing isolates in a phylogeny using SNPs and then discriminating among isolates within SNP-determined subclades using MLVA. This step-wise approach has been termed Progressive Hierarchical Resolving Assays using Nucleic Acids (PHRANA) [24].

## Conclusions

We describe a new subpopulation in the B.Br.013 group from Georgia that is genetically and geographically distinct from the other B.Br.013 group subpopulations found in Europe. Members of this new lineage are endemic to parts of Eastern Europe and Western Asia, though the complete geographic range remains unknown. The basal positioning of the Georgian lineage and its restricted geographic distribution illustrates the need for studies on additional Asian and East European isolates to gain a better understanding of the clonal expansion of *F. tularensis *subsp. *holarctica*.

## Methods

### Whole Genome Sequencing

We sequenced a single Georgian isolate (F0673), representing the most common MLVA profile type of *F. tularensis *subsp. *holarctica *found in the country of Georgia (Chanturia, unpubl. data), using Illumina's Genome Analyzer II (San Diego, CA). DNA from F0673 was prepared using a standard chloroform extraction protocol [34]. Library preparation for this isolate involved sonication of 5 μg genomic DNA to an average fragment size of 350 bp, followed by sample preparation and cluster generation protocols for paired-end reads from Illumina. The library was quantified using SYBR-based qPCR and primers modified from the adaptor sequence. The library was then run in two lanes of the flow cell to increase overall coverage. Read lengths were ca. 40 bp, with a final yield of 32 Gb of sequence for the entire run. Image analysis for base calling and alignments followed the methods of Craig and colleagues [35]. The entire Sequence Read Archive of F0673 was deposited to GenBank (SRP003002.2).

### SNP Discovery and Analysis

To identify putative SNPs, the Georgian isolate WGS was aligned with LVS (*F. tularensis *subsp. *holarctica *LVS NC_007880) and was compared to four other WGSs available from GenBank (*F. tularensis *subsp. *holarctica *FSC 200 NZ_AASP00000000, *F. tularensis *subsp. *holarctica *LVS NC_007880 and *F. tularensis *subsp. *holarctica *OSU18 NC_008369) and the Human Genome Sequencing Center at Baylor College of Medicine (*F. tularensis *subsp. *holarctica *RC503 http://www.hgsc.bcm.tmc.edu/microbial-detail.xsp?project_id=144). Three of these WGSs (FSC 200, LVS, and RC503) were selected because of their membership in the B.Br.013 group, whereas the OSU18 WGS was selected as an outgroup. *F. tularensis *subsp. *tularensis *SCHU S4 (NC_006570) was used for referencing SNP positions. Two independent approaches were used for SNP discovery, the MAQ algorithm [36] and a custom SNP calling pipeline. The in-house pipeline used for SNP discovery first compares WGSs in a pairwise fashion using MUMmer [37] to identify putative SNPs and then uses PERL and Java Scripts for grouping the discovered SNPs by shared location, comparing SNPs across all taxa and tabulating the final putative SNP set according to certain criteria. Specifically, SNPs from repeated regions, including paralogous genes, apparent tri-state SNPs and SNPs with an adjacent SNP closer than 11 bp away were removed from analysis. Furthermore, the SNP locus must be present in all of the genomes to be included in the analysis. The software package PAUP 4.0b10 (D. Swofford, Sinauer Associates, Inc., Sunderland, MA) was used to construct a whole genome SNP phylogeny (Figure 1B) using maximum parsimony.

### CanSNP Selection and Analysis

Thirty-nine putative SNPs specific to the Georgian lineage were identified in the whole genome sequence analysis. Of these, twenty-one were incorporated into melt-MAMA genotyping assays, as previously described [15], except that only GC- rich tails were used on one allele specific primer [38]. A melt-MAMA assay was also designed for branch B.Br.026 within the B.Br.013 group. Allele-specific melt-MAMA primers were designed using Primer Express 3.0 software (Applied Biosystems, Foster City, CA) (Table 1). All other assay reagents and instrumentation were as previously described [15]. DNA templates were extracted using either chloroform [34] or DNeasy blood and tissue kits (Qiagen, Valencia, CA). Reactions were first raised to 50°C for 2 min to activate the uracil glycolase, then raised to 95°C for 10 min to denature the DNA and then cycled at 95°C for 15s and 55°C for 1 min for 33 cycles (Table 1). Immediately after the completion of the PCR cycle, amplicon melt dissociation was measured by ramping from 60°C to 95°C in 0.2°C/min increments and recording the fluorescent intensity. The genome locations, primer sequences and annealing temperatures for the seven canSNP assays can be found in Table 1. We screened a geographically diverse panel of 132 European isolates belonging to the B.Br.013 group and a geographically diverse panel of 25 Georgian isolates across lineage-specific assays to determine whether they were in the B.Br.026 or the Georgian (B.Br.027) lineages (see additional file 3, Table 2).

### MLVA

All 25 Georgian isolates were screened with an 11-marker MLVA system (Additional file 4) [25]. This was done in order to determine the level of genetic diversity within each identified subclade. The MLVA Diversity (D) was calculated for each subclade using the following equation: G/N (G = MLVA genotypes; N = number of isolates). Diversity was not calculated for subclades with a single isolate.

## Authors' contributions

GC and DNB carried out the molecular genetic studies, constructed the figures, performed data analysis, and drafted the manuscript. EZ and GB carried out the molecular genetic studies, MK, NT, ST, PI, JF assisted in the design of the study. SMBS, JSBS, SS, and MDC participated in the computational *in silico *data analyses. JTF sequenced the Georgian strain. MG, AHP, and ELK carried out the molecular genetic studies. AJV participated in the design of the study and drafted the manuscript. JDB and TP drafted the manuscript. DMW assisted in the design of the study and drafted the manuscript. PK participated in the project design, data interpretation and drafted the manuscript. All authors read and approved of the final manuscript.

## Authors' information

GC, MS, National Center for Disease Control and Public Health, Tbilisi, Georgia

DNB, PhD, Northern Arizona University, Flagstaff, Arizona

MK, PhD, National Center for Disease Control and Public Health, Tbilisi, Georgia

EZ, MS, National Center for Disease Control and Public Health, Tbilisi, Georgia

GB, MS, National Center for Disease Control and Public Health, Tbilisi, Georgia

NT, MD, Ph.D., National Center for Disease Control and Public Health, Tbilisi, Georgia

ST, MD, Ph.D., National Center for Disease Control and Public Health, Tbilisi, Georgia

PI, MD, Ph.D., National Center for Disease Control and Public Health, Tbilisi, Georgia

JF, Ph.D., U.S. Army Medical Research Institute of Infectious Diseases, Fort Detrick, Frederick, Maryland

SMBS, PhD, Translational Genomics Research Institute, Phoenix, Arizona

JSBS, BS, Translational Genomics Research Institute, Phoenix, Arizona

SS, MS, Translational Genomics Research Institute, Phoenix, Arizona

MDC, PhD, Translational Genomics Research Institute, Flagstaff, Arizona

MG, DVM, MSc, Veterinary Medical Research Institute, Hungarian Academy of Sciences, Budapest, Hungary

AHP, Northern Arizona University, Flagstaff, Arizona

ELK, Northern Arizona University, Flagstaff, Arizona

JDB, PhD, Northern Arizona University, Flagstaff, Arizona

TP, PhD, Northern Arizona University, Flagstaff, Arizona

JTF, PhD, Northern Arizona University, Flagstaff, Arizona

AJV, PhD, Northern Arizona University, Flagstaff, Arizona

DMW, PhD, Northern Arizona University, Flagstaff, Arizona

PK, PhD, Northern Arizona University, and Translational Genomics Research Institute, Flagstaff, Arizona

## Supplementary Material

Additional file 1 ***Francisella tularensis *canSNP revised SCHU S4 positions**. Provides the updated SCHU S4 genome positions for Melt-MAMA assays published in Vogler et al. 2009. Click here for file

Additional file 2 **Coverage plot of Illumina short sequence reads for Georgian strain F0673 aligned to LVS**. Coverage gaps correspond to duplicated regions that contain pathogenicity islands [26], which were omitted from the WGS SNP analyses. Click here for file

Additional file 3 ***Francisella tularensis *subsp. *holarctica *isolates belonging to B.Br.013 group used in this study**. Lists NAU strain ID, original ID, date, and geographic location of isolates used in this study. Click here for file

Additional file 4 ***Francisella tularensis *MLVA genotype data presented as repeat size**. Click here for file
