# Supplementary figures and images for: Phylogeography of Francisella tularensis subspecies holarctica from the country of Georgia
Source: BMC Microbiol. 2011 Jun 17;11:139. doi: 10.1186/1471-2180-11-139 (PMC3224097; doi:10.1186/1471-2180-11-139)

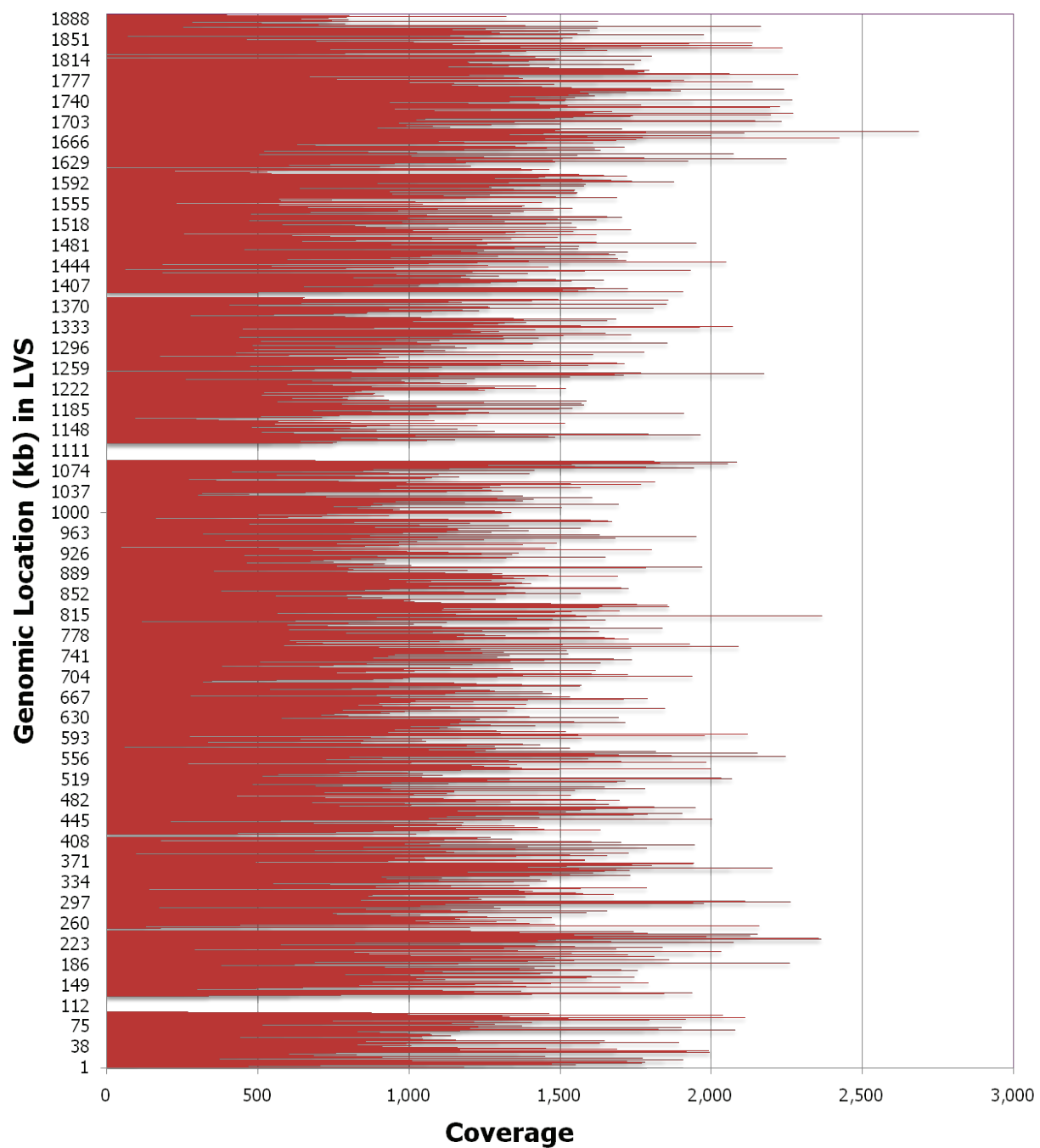

Supplement: Additional file 2 — Coverage plot of Illumina short sequence reads for Georgian strain F0673 aligned to LVS. Coverage gaps correspond to duplicated regions that contain pathogenicity islands [26], which were omitted from the WGS SNP analyses. [file 1471-2180-11-139-S2.PDF]
